# The impact of repeated marathon running on cardiovascular function in the aging population

**DOI:** 10.1186/1532-429X-14-58

**Published:** 2012-08-20

**Authors:** Erin Karlstedt, Anjala Chelvanathan, Megan Da Silva, Kelby Cleverley, Kanwal Kumar, Navdeep Bhullar, Matthew Lytwyn, Sheena Bohonis, Sacha Oomah, Roman Nepomuceno, Xiaozhou Du, Steven Melnyk, Matthew Zeglinski, Robin Ducas, Mehdi Sefidgar, Scott Mackenzie, Sat Sharma, Iain D Kirkpatrick, Davinder S Jassal

**Affiliations:** 1 Institute of Cardiovascular Sciences, St. Boniface Research Centre, University of Manitoba, Winnipeg, MB, Canada; 2 Section of Cardiology, Department of Internal Medicine, University of Manitoba, Rm Y3531, 409 Tache Avenue, St. Boniface General Hospital, Winnipeg, MB, Canada; 3 Section of Cardiac Surgery, Department of Surgery, University of Manitoba, Winnipeg, MB, Canada; 4 Section of Cardiac Anesthesia, Department of Anesthesia, University of Manitoba, Winnipeg, MB, Canada; 5 Sections of Respiratory Medicine and Critical Care, Department of Internal Medicine, University of Manitoba, Winnipeg, MB, Canada; 6 Department of Radiology, University of Manitoba, Winnipeg, MB, Canada

**Keywords:** Marathon running, Cardiac biomarkers, Echocardiography, Cardiac computed tomography, Cardiovascular magnetic resonance

## Abstract

**Background:**

Several studies have correlated elevations in cardiac biomarkers of injury post marathon with transient and reversible right ventricular (RV) systolic dysfunction as assessed by both transthoracic echocardiography (TTE) and cardiovascular magnetic resonance (CMR). Whether or not permanent myocardial injury occurs due to repeated marathon running in the aging population remains controversial.

**Objectives:**

To assess the extent and severity of cardiac dysfunction after the completion of full marathon running in individuals greater than 50 years of age using cardiac biomarkers, TTE, cardiac computed tomography (CCT), and CMR.

**Methods:**

A total of 25 healthy volunteers (21 males, 55 ± 4 years old) from the 2010 and 2011 Manitoba Full Marathons (26.2 miles) were included in the study. Cardiac biomarkers and TTE were performed one week prior to the marathon, immediately after completing the race and at one-week follow-up. CMR was performed at baseline and within 24 hours of completion of the marathon, followed by CCT within 3 months of the marathon.

**Results:**

All participants demonstrated an elevated cTnT post marathon. Right atrial and ventricular volumes increased, while RV systolic function decreased significantly immediately post marathon, returning to baseline values one week later. Of the entire study population, only two individuals demonstrated late gadolinium enhancement of the subendocardium in the anterior wall of the left ventricle, with evidence of stenosis of the left anterior descending artery on CCT.

**Conclusions:**

Marathon running in individuals over the age of 50 is associated with a transient, yet reversible increase in cardiac biomarkers and RV systolic dysfunction. The presence of myocardial fibrosis in older marathon athletes is infrequent, but when present, may be due to underlying occult coronary artery disease.

## Background

Participation in strenuous aerobic physical activity is on the rise in North America, especially among the aging population [1]. The cardiovascular effects of acute strenuous exercise, specifically marathon running, has been studied extensively over the past two decades [2-5]. A number of studies involving marathon participants between the ages of 18 and 40 years, have demonstrated a transient increase in cardiac biomarkers and right ventricular (RV) systolic dysfunction using multimodality cardiac imaging, including transthoracic echocardiography (TTE) and cardiovascular magnetic resonance (CMR) [6-12]. Little is known, however, on the cardiovascular effects of repeated marathon running in individuals over the age of 50.

Although cardiac biomarkers, including cardiac specific TnT, are transiently elevated in participants immediately following the marathon [6-12], it remains unclear whether true myocardial necrosis occurs at the cellular level. Late gadolinium enhancement CMR (LGE-CMR), following administration of gadolinium, has been recently evaluated as a noninvasive method of delineating myocardial necrosis in this patient population [11-16]. In individuals between the ages of 18 and 40, a number of recent marathon studies have demonstrated the absence of LGE of the left ventricular (LV) myocardium [11,12,15,16]. This would suggest that permanent myocardial injury does not occur from repeated marathon running.

On the contrary, Breuckmann et al. recently demonstrated evidence of LGE of the LV myocardium in a heterogeneous cohort of individuals greater than 50 years of age, suggesting myocardial injury may result from the stress of repeated marathon running [14]. In their study, a greater number of participants who ran marathons demonstrated LGE of the LV myocardium when compared to age-matched controls. Although the study excluded runners with a preceding history of ischemic heart disease (IHD), the presence of obstructive coronary artery disease (CAD) was not systematically evaluated. It is entirely plausible that the LGE of the LV myocardium observed in the runners greater than 50 years of age in their patient population may have been due to underlying occult obstructive CAD, rather than as a direct result of repetitive marathon running.

The aims of the current study were two-fold: 1) To assess the extent and severity of cardiac dysfunction after the completion of full marathon running in elite individuals >50 years of age using cardiac biomarkers, TTE and CMR; and 2) If there is evidence of LGE on CMR, to detect the presence of silent coronary artery disease using cardiac computed tomography (CCT).

## Methods

### Study population

A prospective study was performed on 25 consecutive elite individuals who participated in the 2010 and 2011 Manitoba Full Marathons. Subjects over the age of 50 who participated in greater than three marathons in the past two years, were included in the study. Exclusion criteria included a history of smoking, hypertension, elevated lipids, diabetes, and/or contraindications for CCT or CMR. The study protocol was approved by the local institutional review board.

### Cardiac biomarkers

The cardiac biomarkers measured included myoglobin, creatinine kinase (CK), and cardiac specific troponin T (cTnT). These biomarkers were measured at three separate time points: (1) 1 week prior to the marathon; (2) immediately after completion of the full marathon; and (3) 1 week following the marathon. Myoglobin levels were determined using a Roche^TM^ Elecsys analyzer and CK levels were determined using a Roche^TM^ 917 analyzer. An increase in myoglobin and CK levels four times greater than baseline was considered elevated. A third generation Roche Elecsys assay was used to perform quantitative determinations of cTnT levels.

### Echocardiography

All subjects underwent baseline TTE one week prior to the marathon, immediately after completion of the marathon and one week post marathon. Parasternal and apical views were obtained using a standard echocardiography machine (GE Vivid 7, Milwaukee, Il, USA) with a multifrequency transducer and tissue Doppler capability. Standard 2-dimensional images, M-mode, spectral and color Doppler, and tissue Doppler imaging (TDI) were performed.

Interventricular septal thickness (IVS), posterior wall thickness (PWT), left ventricular ejection fraction (LVEF), and left atrial (LA) size indexed to body surface area were determined from 2-dimensional images [17]. Left ventricular mass was calculated using the area-length method per the American Society of Echocardiography guidelines [17]. Right ventricular cavity dimensions, RV fractional area change (FAC) and tricuspid annular plane systolic excursion (TAPSE) were determined [18]. Continuous-wave Doppler was used to measure the peak velocity across the tricuspid valve and the maximal peak pressure gradient was estimated using the simplified Bernouilli equation with addition of the right atrial pressure to calculate the pulmonary artery systolic pressure (PASP) [18]. Transmitral LV filling velocity at the tips of the mitral valve leaflets were obtained from the apical 4-chamber view using pulsed wave Doppler echocardiography. Tissue Doppler derived indices were recorded at the lateral mitral annulus of the LV and the lateral tricuspid annulus of the RV.

### Cardiac computed tomography

All patients underwent CCT using a 64 detector-row GE Lightspeed VCT scanner (General Electric Medical Systems, Milwaukee, Wi, US), within three months of completing the full marathon. A prospective ECG-gated (SnapShot Pulse, GE Medical) CT coronary angiogram was performed. Images were obtained with a tube rotation time of 0.35 sec, 120 kV and 0.625 mm thick contiguous images through the coronary arteries with a field of view to cover the heart (Cardiac Medium, GE Medical) and cardiac phase coverage of 70-80%. All images were reviewed on a 3D workstation (GE Advantage, General Electric Medical Systems, Milwaukee, Wi, US) equipped with a dedicated cardiac CT software package (SmartScore and CardIQ Express, GE Medical). Source images for the CT angiogram were analyzed along with multiplanar reformations, curved planar reformations of each coronary artery, and double oblique reformations of each vessel axial to the lumen throughout its course.

### Cardiovascular magnetic resonance

CMR was performed on all study participants at baseline and within 12 hours following completion of the full marathon using a 1.5-T scanner (Avanto; Siemens Medical Solutions, Erlangen, Germany). Breath-hold cine imaging was performed using a segmented TrueFISP sequence with ECG gating to achieve 25 images covering the entire cardiac cycle. To evaluate for myocardial edema, IR-prepared dark blood T2-weighted turbo spin echo short axis images were obtained (TR 1800–2100 ms, TE 74 ms, 8 mm slice thickness, 4 mm interslice gap, matrix 256 X 175). Late gadolinium enhancement images were obtained after 10 minutes of 0.2 mmol/kg injection of Gadolinium (Gd-DTPA, Magnevist, Schering, Germany) using a T1-weighted IR-prepared multislice TurboFLASH sequence with magnitude and phase sensitive reconstruction. Images were acquired sequentially in the short axis, followed by horizontal and vertical long axis images (TR 700 ms, TE 3.36 ms, FA 25°, 8 mm slice thickness, 1.6 mm interslice gap, matrix 256 × 192). The CMR images were analyzed using CMR^42^ (Release 2.2.0, Circle Cardiovascular Imaging, Calgary, Alberta, Canada). Endocardial and epicardial contours were drawn manually for the LV and RV, respectively, at end-systole and end-diastole in each data set with the most basal short axis slice identified as the image which contains at least 50% of circumferential myocardium. Papillary muscles and trabeculations were included in LV and RV mass calculation. All TTE, CCT and CMR images were analyzed by two experienced reviewers (IK and DJ) blinded to the clinical data.

### Statistics

The data are summarized as mean ± SD, number (percentage), or median and interquartile range. Paired Student’s t-tests were used to compare continuous variables. Chi-square and Fisher’s exact tests were applied to compare categorical variables. One-way analysis of variance (nonparametric with Dunn testing) was used to compare baseline, immediate, and one week post marathon cardiac biomarkers and echocardiographic values. A p-value <0.05 was considered statistically significant. SAS version 9.02 (SAS Institute Inc., Cary, North Carolina) was used to perform the analysis.

## Results

In 2010 and 2011, a total of 1393 individuals (998 males) participated in the Full Manitoba Marathons, completing the race with an average time of 259 ± 42 minutes. Our study population included 25 individuals (21 males, 55 ± 4 years) over the age of 50 who had completed 3 or more marathons in the past two years, with a mean 57 ± 8 marathons participated to date. All patients were moderately to highly trained with a mean 47 ± 7 miles/week. The mean completion time of our study population was 252 ± 33 minutes. The weights, heights and body mass indices of the subjects did not change significantly after the full marathon (Table 1).

**Table 1:** Patient clinical characteristics (n = 25)

**Characteristics**	**Baseline**	**Post-race**
Age (y)	55 ± 4	
Gender, n (%)		
Male	21 (84)	
Female	4 (16)	
Weight (kg)	68±9	68±8
Height (cm)	169±8	169±8
BMI (kg/m^2^)	24±2	24±2
Heart rate (bpm)	63±11	95±8
SBP (mm Hg)	128±11	114±12
DBP (mm Hg)	72±5	67±6

Values are mean ± SD. y, years; *BMI,* body mass index; bpm, beats per minute; *SBP*, systolic blood pressure*; DBP,* diastolic blood pressure.

At baseline, one week prior to the marathon, plasma levels of cardiac biomarkers including myoglobin, CK and cTnT were all within normal limits. Subsequently, each patient demonstrated a significant increase in each biomarker level immediately following the marathon (Table 2). Myoglobin levels increased from a median of 42 mg/L at baseline to 690 mg/L immediately following the marathon. CK levels increased from 125 U/L at baseline to 752 U/L. Finally, cTnT levels increased from <0.01 μg/L at baseline to a median of 0.52 μg/L immediately after the race. One week post marathon, myoglobin, CK and cTnT levels returned to baseline values (Table 2).

**Table 2:** Cardiac biomarker data for study population at baseline and post marathon (n = 25)

**Characteristics**	**Baseline**	**Immediately post race**	**1 wk post marathon**
Myoglobin (mg/L)	42 (27–90)	690 (416–1832)*	72 (50–101)
CK (U/l)	125 (96–185)	752 (445–1829)*	165 (103–375)
cTnT (ug/L)	<0.01	0.52 (0.38-0.81)*	<0.01

Data are expressed as median (interquartile range).

*p < 0.05, after the race vs. baseline.

Using TTE and CMR, RV structure and function changed significantly from baseline to immediately after the marathon (Tables 3 and 4). There was an increase in right atrial volume and RV end-diastolic diameter, with a decrease in RV FAC and TAPSE immediately post marathon (Table 3). The RV TDI parameters including S’ and E’ decreased immediately following the race and remained abnormal at one week of follow-up (Table 4). Similarly, CMR demonstrated an in increase in RV end-diastolic volume and a decrease in RVEF following the marathon (Table 5). There was an increase in peak pulmonary arterial systolic pressure from 14 ± 3 mm Hg at baseline to 44 ± 6 mm Hg following the race. At one week post marathon, RV volumes and function by both TTE and CMR had returned to baseline values.

**Table 3:** Echocardiographic data in study population at baseline and post marathon (n = 25)

**Echo parameters**	**Baseline**	**Post-race**	**Follow-up**	**p-value**
** *LV parameters (2D TTE)* **				
LVEDD (mm)	50 ± 3	51 ± 5	50 ± 4	0.93
LVESD (mm)	33 ± 5	34 ± 4	32 ± 5	0.82
LVEDV (ml)	114 ± 12	110 ± 10	112 ± 11	0.72
LVESV (ml)	39 ± 11	38 ± 13	40 ± 11	0.77
IVS (mm)	9 ± 2	9 ± 1	9 ± 2	0.82
PWT (mm)	9 ± 1	8 ± 2	9 ± 2	0.71
LVEF (%)	63 ± 4	62 ± 6	64 ± 3	0.79
LV mass/BSA (g/m^2^)	102 ± 11	103 ± 15	99 ± 17	0.49
** *LA parameters (2D TTE)* **				
LA diameter (mm)	36 ± 4	37 ± 3	36 ± 5	0.66
LA volume (ml)	42 ± 11	44 ± 13	40 ± 13	0.44
** *RA and RV parameters (2D TTE)* **				
RA volume (ml)	37 ± 12	61 ± 12	31 ± 13	0.01*
RVEDD (mm)	27 ± 3	45 ± 2	32 ± 4	0.02*
RV FAC (%)	48 ± 3	29 ± 6	49 ± 4	0.01*
TAPSE (mm)	2.3 ± 0.4	1.4 ± 0.2	2.1 ± 0.3	0.02*

Data are expressed as mean ± SD. *p < 0.05, after the race vs. baseline.

2D, 2 dimensional; TTE, transthoracic echocardiography; LVEDD, LV end-diastolic diameter; LVESD, LV end-systolic diameter; LVEDV, LV end-diastolic volume; LVESV, LV end-systolic volume; IVS, interventricular septum; PWT, posterior wall thickness; LVEF, LV ejection fraction; BSA, body surface area; LA, left atrium; RA, right atrium; RVEDD, RV end-diastolic diameter; FAC, fractional area change; TAPSE, tricuspid annular plane systolic excursion.

**Table 4:** Conventional and novel diastolic echo parameters in patient population (n = 25)

**Echo parameters**	**Baseline**	**Post-race**	**Follow-up**	**p value**
** *Doppler echocardiography* **				
Mitral E velocity (cm/s)	0.8±0.2	0.5±0.3	0.8±0.2	0.76
Mitral A velocity (cm/s)	0.5±0.1	0.5±0.2	0.5±0.2	0.68
Mitral E/A ratio	1.6±0.2	1.5±0.1	1.6±0.1	0.72
Mitral E decel time (ms)	208±52	212±63	214±48	0.65
** *Left Ventricle: Tissue Doppler imaging* **				
Lateral S’ (cm/s)	10.1±1.0	10.2±0.3	10.4±1.0	0.58
Lateral E’ (cm/s)	11.4±0.6	7.6±1.1^*^	10.1±0.7^**^	<0.01
Lateral A’ (cm/s)	4.2±1.2	8.2±1.0^*^	6.1±1.1^**^	<0.01
** *Right Ventricle: Tissue Doppler imaging* **				
S’ at base (cm/s)	11.3±0.9	8.7±1.2^*^	11.0±0.9	<0.01
E’ at base (cm/s)	11.6±1.0	9.5±0.9^*^	10.3±0.5^**^	<0.01
A’ at base (cm/s)	7.8 ±1.1	10.3±0.9^*^	9.8±0.4^**^	<0.01

Values are mean ± SD. *MR*, mitral regurgitation; decel, deceleration; *PASP*, pulmonary artery systolic pressure. ^*^Post-race compared to baseline. ^**^One week follow-up compared to baseline.

**Table 5:** CMR data in study population at baseline and 24 hr post marathon (n = 25)

**CMR parameters**	**Pre marathon**	**Post race**
** *LV parameters* **		
LVEDD (mm)	52 ± 3	51 ± 4
LVESD (mm)	31 ± 4	30 ± 5
LVEDV/BSA (ml/m^2^)	82 ± 9	84 ± 7
LVESV/BSA (ml/m^2^)	24 ± 8	26 ± 6
IVS (mm)	9 ± 1	9 ± 1
PWT (mm)	9 ± 2	9 ± 1
LVEF (%)	67 ± 4	69 ± 3
LV mass/ BSA (g/m^2^)	126 ± 14	123 ± 9
** *LA parameters* **		
LA diameter (mm)	34 ± 4	36 ± 6
LA volume/ BSA (ml/m^2^)	26 ± 8	27 ± 4
** *RA and RV parameters* **		
RA volume (ml)	39 ± 8	57 ± 10^*^
RVEDD (cm)	33 ± 5	47 ± 4^*^
RVEDV (ml)	133 ± 19	190 ± 18^*^
RVEF (%)	65 ± 3	44 ± 6^*^
RV mass/BSA (g/m^2^)	32 ± 4	34 ± 3

Data are expressed as mean ± SD. *p < 0.05, after the race vs. baseline.

CMR, cardiac magnetic resonance; LVEDD, LV end-diastolic diameter; LVESD, LV end-systolic diameter; LVEDV, LV end-diastolic volume; LVESV, LV end-systolic volume; IVS, interventricular septum; PWT, posterior wall thickness; LVEF, LV ejection fraction; BSA, body surface area; LA, left atrium; RA, right atrium; RVEDD, RV end-diastolic diameter; RVEDV, RV end-diastolic volume; RVEF, RV ejection fraction.

**Figure 1:**
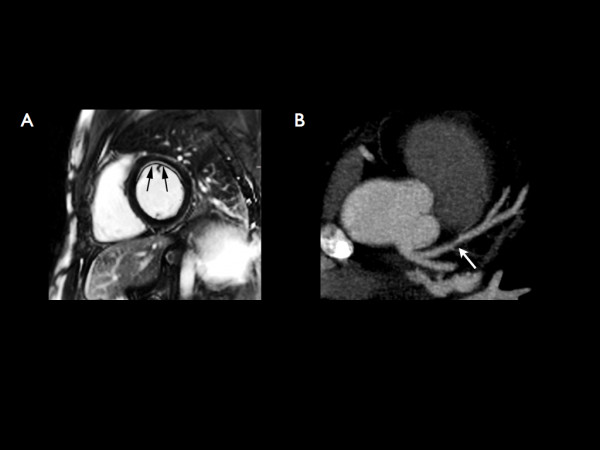
**A) Short axis phase sensitive IR-prepared T1-weighted TurboFLASH MR image demonstrating subendocardial enhancement involving the anterior wall of the LV at baseline, prior to the marathon (arrows).B**) Curved planar reformation of a contrast-enhanced CT angiogram in the same patient demonstrating a >70% stenosis of the LAD artery (arrow).

Of the total study population, 23 individuals demonstrated no evidence of myocardial edema and no LGE of the LV myocardium using CMR, either before or after the marathon. There was no evidence of obstructive CAD on CCT in these 23 subjects. Two participants, however, demonstrated LGE of the sub-endocardial layer of the anterior wall of the LV at baseline, with no change following completion of the marathon (Figure 1A). The corresponding CCT in these two individuals demonstrated greater than 70% stenosis of the left anterior descending artery (Figure 1B).

## Discussion

Our study confirmed a transient but reversible increase in cardiac biomarkers in individuals over the age of 50, following completion of a full marathon. While LV structure and function was not altered acutely following strenuous exercise, we observed a decrease in RV systolic function confirmed by both TTE and CMR, which normalized one-week post marathon. There was no evidence of LGE of the LV on CMR in the majority of study participants, suggesting that permanent myocardial injury does not occur due to the stress of repeated marathon running in older individuals. In the two individuals with LGE of the LV in a subendocardial distribution at baseline, the myocardial injury was most likely due to underlying CAD.

Multiple studies have demonstrated a transient increase in cardiac biomarkers of injury and acute RV systolic dysfunction following marathon running in younger individuals (18–40 years) [6-9,11,12,15]. While increased RA and RV dimensions and decreased RV systolic function has been confirmed on both TTE and CMR in younger marathon participants, little is known about the effects of endurance running in older participants. In a 2009 study by Knebel et al., 78 male marathon runners were evaluated using both cardiac biomarker analysis and TTE [1]. The study population was divided into older (>60 years; n = 23) and younger (<60 years; n = 50) individuals. In the older cohort, nearly 30% demonstrated an increase in cTnT and N-terminal pro-brain natriuretic peptide (NT-proBNP) immediately after the race, which normalized 2-weeks post marathon [1]. In our study, all participants demonstrated an abnormal biochemical profile immediately after completing the marathon, confirming the findings of Knebel et al [1]. They also demonstrated that TDI strain analysis of the basal, mid and apical RV free wall using TTE decreased after prolonged exercise in the older cohort, indicative of RV functional impairment [1]. Using both TTE and CMR, our study also confirms transient RV systolic dysfunction due to exercise induced pulmonary hypertension in an older cohort of endurance athletes [1,19].

While there is significant agreement between studies regarding the elevation of cardiac biomarkers immediately post-marathon, its cause remains unclear [6-9,16,20-25]. LGE-CMR imaging is used routinely for the noninvasive detection of myocardial fibrosis. The pattern of LGE allows for the differentiation of CAD versus non-CAD etiologies of myocardial scar formation. Four previous studies using a younger cohort of marathon athletes demonstrated no evidence of LGE of the LV myocardium, suggesting that true myocardial necrosis does not occur [11,12,15,16]. Our current study extends this understanding to an older cohort of individuals, whereby repeated endurance stress does not seem to result in myocardial fibrosis in this patient population.

Recently, Breuckmann et al. demonstrated that marathon participants exhibited LGE of the LV myocardium three times more often than their age-matched controls [14]. They also noted different patterns of LGE within the marathon participant group; 42% exhibited LGE typical of a prior myocardial infarction, while the remaining 58% exhibited LGE in an atypical pattern described as patchy or streaky [14]. While a preceding history of IHD was an exclusion factor for participation in their study [14], the presence of occult CAD was not systematically evaluated. These findings were recently confirmed by a smaller study by Wilson et al. who observed LGE in 6 of 12 elite veteran athletes (57 ± 6 years), but not in 17 younger (31 ± 5 years) athletes [26]. Although the LGE pattern was described in a non-CAD pattern in 5 of these 6 older athletes, the underlying cause of this observation was not evaluated [26].

Our study is the first to systematically evaluate the presence of occult CAD using CCT as a potential cause of myocardial fibrosis in marathon athletes over the age of 50. Two individuals were clinically asymptomatic, but were found to have LGE of the anterior wall of the LV myocardium in a subendocardial distribution prior to running the marathon, with concomitant evidence of obstructive LAD disease. In a 2008 study by Mohlenkamp et al., recreational marathon runners presenting with myocardial LGE were found to have higher coronary artery calcification (CAC) scores [2]. Their study findings supported a pathophysiological link between epicardial subclinical plaque burden and myocardial damage in marathon runners, increasing our awareness of CAD in this patient population [2].

Although the presence of LGE of the myocardium in older endurance marathoners may be due to underlying CAD, the small number of individuals in our study does not allow for any definitive conclusions. A larger patient population from all ages with both CCT and CMR would allow for defining the prevalence of silent CAD in marathon participants. Future studies addressing the long-term cardiovascular outcomes of athletes with incidental findings of CAD and LGE are required.

## Conclusion

Marathon running in individuals over the age of 50 is associated with a transient, yet reversible increase in cardiac biomarkers and RV systolic dysfunction. The presence of myocardial fibrosis in older marathon athletes is infrequent, but when present, may be due to underlying occult CAD. Larger studies are needed to confirm these findings.

## Competing interests

The authors declare that they have no competing interests.

## Authors’ contributions

All authors contributed to the writing of the manuscript. All authors read and approved the final manuscript.
